# Impact of Nutrition on Non-Coding RNA Epigenetics in Breast and Gynecological Cancer

**DOI:** 10.3389/fnut.2015.00016

**Published:** 2015-05-27

**Authors:** Rosanna H. E. Krakowsky, Trygve O. Tollefsbol

**Affiliations:** ^1^Department of Biology, University of Alabama at Birmingham, Birmingham, AL, USA; ^2^Department of Biochemistry, University of Leipzig, Leipzig, Germany; ^3^Comprehensive Center for Healthy Ageing, University of Alabama at Birmingham, Birmingham, AL, USA; ^4^Comprehensive Cancer Center, University of Alabama at Birmingham, Birmingham, AL, USA; ^5^Nutrition Obesity Research Center, University of Alabama at Birmingham, Birmingham, AL, USA; ^6^Comprehensive Diabetes Center, University of Alabama at Birmingham, Birmingham, AL, USA

**Keywords:** epigenetics, diet, microRNA, non-coding RNA, female cancer

## Abstract

Cancer is the second leading cause of death in females. According to the American Cancer Society, there are 327,660 new cases in breast and gynecological cancers estimated in 2014, placing emphasis on the need for cancer prevention and new cancer treatment strategies. One important approach to cancer prevention involves phytochemicals, biologically active compounds derived from plants. A variety of studies on the impact of dietary compounds found in cruciferous vegetables, green tea, and spices like curry and black pepper have revealed epigenetic changes in female cancers. Thus, an important emerging topic comprises epigenetic changes due to the modulation of non-coding RNA levels. Since it has been shown that non-coding RNAs such as microRNAs and long non-coding RNAs are aberrantly expressed in cancer, and furthermore are linked to distinct cancer phenotypes, understanding the effects of dietary compounds and supplements on the epigenetic modulator non-coding RNA is of great interest. This article reviews the current findings on nutrition-induced changes in breast and gynecological cancers at the non-coding RNA level.

## Introduction

Current research provides evidence for the importance of nutrition in terms of health and disease prevention via phytochemicals and their modes of action. Studies on the “epigenetic diet” have revealed that the consumption of soy, curry spices, red grapes, as well as blueberries has beneficial effects on the prevention of diseases like cancer (1, 2). This applies not only to the individual consuming the epigenetic diet but also applies to the exposure of the unborn child *in utero*, which can lead to reprograming of the existing epigenetic profiles and hence changes the predisposition to diseases like cancer (2). One way of exerting its impact is through modulation of non-coding RNA levels, microRNAs (miRs) in particular, in the individual (3, 4). miRs are endogenous RNAs of 18–25 nucleotides in length that regulate the expression of genes through binding to the 3′UTR of mRNA, and thus leading to the degradation of the bound mRNA or to the inhibition of translation. Furthermore, it was shown that miRs can also target the 5′UTR, promoter, or coding sequences (5, 6).

Since miRs can either modulate or serve as tumor suppressors or oncogenes, two scientific approaches toward targeting cancer are under current investigation. One approach is to utilize miRs such as let-7, miR-34, and miR-29 in patients for anticancer therapy. The other is employing miR levels as biomarkers to diagnose, classify, and predict the clinical outcome of cancer patients. Due to the fact that circulation of miRs in blood, milk, urine, and various other body fluids has been frequently proven, their employment as biomarkers is self-evident (7). Along with those findings, the detection of microvesicles containing miR being released only from breast cancer (BC) cells further underlines the importance of investigating miRs in the circulation (8). A goal is to profile miRs to enable personalized therapies and treatment of patients in the future, and elucidating the impact of phytochemicals on miRs is one important aspect of this individualized medicine.

## Overview of miR Biogenesis

The genesis of miRs is a multistep process and eventually leads to the alteration of protein levels by RNA silencing. During this process, RNA polymerase II transcribes the primary transcript, pri-miR, from inter-or intragenic regions. The recognition and cleavage of the primary transcript through Drosha, the microprocessor complex and DiGeorge critical region 8 (DGCR8), follows thereafter (7). Furthermore, an alternative pathway independent of Drosha digestion spawning “miRtrons,” functional pre-miRs which are derived from spliced out mRNA introns, has been described (7). While Exportin-5, GTP, as well as Ran, promote the release of the pre-miR from the nucleus into the cytoplasm by recognizing its 3′ 2-nucleotide (nt) overhang, Dicer, a further RNase III, digests the pre-miR, creating an approximately 22 nt-long miR duplex (7). Common for mammals, the two miR strands differ in their thermodynamical stability, and the guide strand with the less stable 5′-end serves as template for target recognition in the RNA-induced silencing complex (RISC) (9). Until recently, the remaining strand was thought to be degraded; however, there has been evidence suggesting that the miR*-passenger strand levels are in accordance with their corresponding guide strand levels. It remains unclear whether the guide strand only or the passenger strand also may be incorporated into the RISC (9). To date, the guide strand, also referred to as miR-5p form, is thought to be more active due to its higher abundance than the miR*-passenger strand (miR-3p form), which shows a higher probability of degradation. Yet, the relative expression of miR-5p and miR-3p remains undiscovered (10).

The RISC, consisting of the transactivation-responsive RNA binding protein and the catalytic component Argonaute, elicits its action by complementary binding of the 3′UTR mRNA target through the incorporated miR. Either translational repression or mRNA cleavage results from mRNA recognition by the RISC. In case of inaccurate complementary binding, translational repression occurs, whereas in the case of accurate complementarity mRNA cleavage is brought about by Argonaute. Subsequently, a decrease in protein levels is effectuated (11). The level of suppression of protein biosynthesis is thus dependent on the amount of expressed miR; overexpressed miR leads to under-expressed target genes and vice versa. In this manner, miRs are able to modulate pathways and multiple downstream targets involved in cell proliferation, cell cycle progression, apoptosis as well as invasion, migration, and differentiation (5).

## miR Profiling of Female Cancers

Tumor tissue miRs are commonly studied because particular expression patterns can be related to certain phenotypes and cancer properties. However, the miR pattern is unique to the cancer type in general as well as to the individual cancer tissue, hence, making broad generalizations is met with challenges. This also indicates that the same miR may act as tumor suppressor in one cancer type, whereas it may act as an oncogene in another. An epitome of this concept is miR-93. While being overexpressed in many cancer cases, miR-93 has also been found to have tumor suppressor properties. In less differentiated BCs, increased miR-93 expression halts tumor development and metastasis; whereas its up-regulation in more differentiated tumors was observed to result in an augmented stem cell population (12, 13). Nevertheless, there are commonly described down-regulated miR families with tumorsuppressor function (oncosuppressormiRs) in female cancers. Examples are let-7, miR-200, as well as the miR-34 family. Oncogenic miRs (oncomiRs) comprise for instance miR-155, miR-27a, miR-21, and miR-221/222.^1^ Noteworthy at this point is the variety of functions of the miR-221/222 cluster. Like miR-93, this cluster shows oncogenic features by targeting PTEN, p27/Kip1, p57, and TIMP3 in BC and other cancer types. But when down-regulated in erythroblastic leukemia, it targets the KIT oncogene (14).

**Figure 1:**
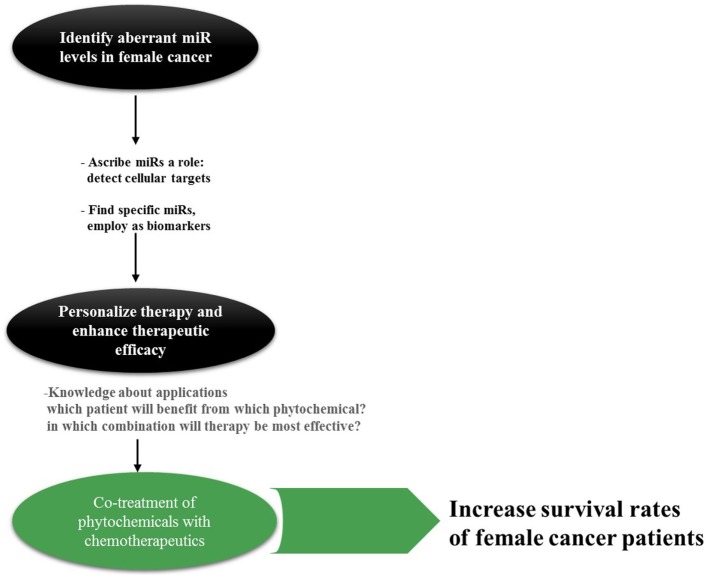
**Rationale behind miR research in cancer**. Especially for cancer types such as OC with its non-specific symptoms, specific and reliable biomarkers are actively sought for. miRs can be found in a variety of body fluids; hence, detection and surveillance of cancer development as well as assigning it into groups may be accomplished in a non-invasive manner in the future. Knowledge about the appropriate application of phytochemicals is inevitable for this approach; however, only little is established today.

Taken together, miRs act in complex biological networks that are not yet fully understood, nevertheless, comprehending these networks and their contribution to emerging female cancers is of great importance. When administering chemotherapy, the different miR profiles of female cancers have to be taken into account as there have been indications of the impact of miRs on the response to treatment, especially on drug resistance (15). Hence, the understanding of miR profiles in female cancers is essential before aiming at reversing their aberrant expression by compounds such as phytochemicals. Despite the obstacles of ambivalent miR functions and expression patterns, the classification of tumors as well as the mapping of miR genes and their regulatory sequences can provide critical information about the tumor (16). Going beyond identification of miR clusters through RT-PCR and microarray techniques is the first step, finding the mechanisms of dysregulation and potential targets the next toward improving survival rates of female cancers (Figure 1).

### miR profiles in breast cancer – an insight

According to Cancer Research UK, BC is the second leading cause of death among cancer cases. While the most common BC is ductal carcinoma, variable factors including environmental, lifestyle, and genetic factors are associated with BC risk. Despite the exposure to Bisphenol A (BPA) as well as age factors, the National Cancer Institute specifies that the risk of developing BC is critical to the inheritance of BRCA1 or BRCA2 mutations, as 55% of women are carriers of those altered tumor suppressor genes and will develop BC. In this connection, miR-146a and miR-146b-5p were discovered to influence BRCA1 by down-regulating it in sporadic triple negative breast cancer (TNBC) (17).

In correspondence with the specific BC subtypes, distinct miR patterns that made it possible to distinguish between basal and luminal subtypes in an independent subset of data were reported (Table 1). For example, miR-18a belonging to the miR-17-92 cluster as well as members of the paralog miR-106b-25 cluster were found to be significantly higher expressed in grade 3 basal-like BC, whereas the let-7 family was significantly lower-expressed in this subtype (18, 19). A preliminary study in TNBC biopsies endorsed that a comparatively high expression of miR-200b-3p, miR-109a, and lower miR-512-5p expression may be linked to an improved response to chemotherapy. Due to the low amount of samples, however, only a trend could be observed (20).

**Table 1:** **Exemplary dysregulated miRs in female cancers and their indications**.

Cancer type	miR	Regulation	Indications	Diagnostic, predictive, prognostic markers	Reference
BC	miR-9	↑ N-Myc and c-Myc induced miR expression	Targets E-cadherin, thus facilitating migration and invasion	Local recurrence and estrogen receptor status	(21, 22)
	miR-17-92 cluster	↑ Myc binds to E-box of first intron of miR-17-92 gene	Amplified in BC	Pancreatic cancer (miR-18); CLL (miR-20a); significantly higher expressed in grade 3 basal-like BC	(18, 19, 21)
	miR-93	↑	Halts tumor development in less diff. BC; in more diff., tumors increase in BCSC	Highly expressed in high grade tumors; differentiation between cancer and cancer-free controls	(18, 23)
	let-7	↓ Wnt-β-catenin pathway represses let-7 expression by transactivating Lin28	let-7 family members target large gene quantity; let-7a suppresses migration and invasion of BC by down-regulating C–C chemokine receptor type 7	Down-regulated in BC with high proliferation index/lymph node metastasis	(24–26)
	miR-200c	↓	Targets BMI1, suppresses clonal expansion of cancer cells and formation of mammary ducts by normal mammary stem cells; tumor formation, inhibits metastasis of BC through targeting HMGB1; positive impact on Dicer levels	BC progression	(27–29)
	miR-221/222 cluster	↑	miR-221 facilitates tumorigenesis in TNBC; miR-221 targets p27	miR221/222 induced repression of Dicer in ERα− BC, ERα status linked to miR221/222 cluster	(28, 30)
	miR-146a, miR-146b-5p	↑	Decrease *BRCA1* expression in sporadic TNBC	miR-146a levels sigificantly increased in plasma of BC patients; basal-like breast tumours decreasedly epxress *BRCA1*	(17, 31)
	miR-34a	↓	Suppresses proliferation and migration of BC by decreasing levels of Bcl-2 and SIRT1	Poor prognosis demonstrated in three independent cohorts of primary BC	(32, 33)
	miR-21	↑	Correlated with advanced clinical stage, lymph node metastasis, and poor prognosis	Serum miR-21 high diagnostic accuracy for BC patients	(34, 35)
	miR-155	↑	Overexpression is associated with metastasis	Poor prognosis	(36)
	miR-497	↓	Inhibts cell growth, migration, and invasion; targets cyclin E1	Associated with higher differentiation grade, positive HER-2 expression, higher incidence of lymph node metastasis, and advanced clinical stage	(37–39)
	miR-205	↓ In TGF-β or Pez induced cells that consequently were subjected to EMT	EMT by targeting ZEB1 and SIP1; targets also ERBB2 and VEGF-A; directly targets HER3 receptor and inhibits the activation of the downstream mediator AKT	miR-205 reduced in tumor probes compared to according normal probes of mammary ducts and lobules	(40–43)
	miR-210	↑ Induced by hypoxia (HIF and VHL)	Involved in hypoxia pathway	Predictive effect on poor survival; higher risk of recurrence and metastasis, thereunder ER−, lymph-node negative cancers	(44–46)

OC	miR-9	↓	Inhibits talin 1/FAK/AKT pathway; sensitises ovarian xenograft tumors to cisplatin and PARP inhibitors	Acts as tumorsuppressor in recurrent OC	(47–49)
	miR-497	↓	Represses pro-metastatic factor SMURF1	Shorter overall survival in patients with serous cystadenoma	(39)
	miR-21	↑ Increased via JNK-1/c-Jun pathway in cisplatin resistant OC cell lines	Targets PDCD4, induces cell growth, inhibition of miR-21 leads to apoptosis and chemosensitivity in OC; miR-21-3p increases cisplatin resistance thorugh targeting NAV3 in OC cell lines	NAV3 is repressed in OC tumors resistant to platinum treatment	(50–52)
	let-7 family	↓ ↑	Let-7g sensitises ADR-RES cells to taxol/vinblastine by down-regulating IMP-1 and MDR1; let-7 targets HMGA2	Increased expression of let-7b correlated with poor prognosis in high grade serous OC; in further study of serous OC, decreased let-7b expression was associated with poor prognosis; loss of let-7 expression in less differentiated cancer types	(53–56)
	miR-200s	↓ ↑	PTEN repression, proliferation, metatstasis *in vitro*; miR-200c can inhibit tumorigenicity and metastasis in CD117+CD44+ OC stem cells and can restore of chemosensitivity in xenograft model	Low miR-200c is associated with poor prognosis	(57–59)
	miR-221	↑	Represses p27 and p57	Serum miR-221 up-regulated in EOC patients	(60–62)
	miR-27a	↑ In ALDH1+ and chemoresistant cells	Associated with chemoresistance	High in a patient subgroup with very poor prognosis	(63, 64)
	miR-210	↑ In response to hypoxia; up-regulated upon VHL inactivation	Facilitates tumor growth through targeting PTPN1 and repressing apoptosis *in vitro*; loss of miR-210 function may cause deregulation of hypoxia response in cancer cells	n/a	(65–67)
	miR-145	↓	Targets Sp1 and Cdk6, sensitizes OC to Paclitaxel; overexpression is linked to inhibition of proliferation, invasion, and apoptosis	OC patients have low levels of miR-145 in serum and tissue	(68, 69)
	miR-205	↑	Facilitates proliferation and invasion of OC	Together with CA-125 and let-7f, high diagnostic accuracy for EOC; elevated plasma levels in cancer patients versus control patients	(70, 71)
	miR-214	↑	Targets p53/Nanog axis in OC stem cells; targets PTEN thus inducing cell survival/cisplatin resistance; suppresses RNF8	Presence of miR-214 in exosomes as well as in according tumor samples	(72–75)

UC	miR-205	↑	Targets PTEN, inhibits apoptosis	Patients with decreased miR-205 expression showed better survival compared to patients with a high miR-205 level	(76, 77)
	miR-34a	↑ In uterine leiomyoma and endometrioid endometrial adenocarcinoma	Inverse correlation between L1CAM and miR-34a levels in endometrial cancer cell lines	Primary tumor sections with increased L1CAM expression showed decreased miR-34a expression; overexpression in endometrioid endometrial adenocarcinoma linked to tumor progression and lymph node involvement	(78–80)
	miR-200 family	↑	200b up-regulates *MMP2* activity by targeting *TIMP2*, 200c targets ZEB1/2, VEGFA, FLT1, IKKβ, KLF9, FBLN5, and TIMP 2, 200c epxression changes upon transition into cancerous cells; reintroduction of miR-200c lowers aggressiveness (migration and invasion) of endometrial cancer cells; miR-200b/c and 429 induce cisplatin resistance by inhibiting AP-2α expression	Compared to normal endometrium, in all EAC stages examined increased; combination of miR-205 and miR-200a predicted relapse; miR-200c ranked prognostic marker for overall survival of endometrioid endometrial carcinoma patients; SNP (rs1045385) possible prognostic marker for cisplatin treatment, as miR binding to AP-2α reduced	(81–86)
	miR-21	↑	Targets PTEN protein expression, thus affecting proliferation	Up-regulated in uterine leiomyoma cohorts, however, n/a for UC patients	(78, 87, 88)
	miR-503	↓	Targets cyclin D1	Patients with relatively higher level improved survival	(89)
	miR-199a-3p, miR-199b	↓	Target mTOR	miR-199b may serve as marker for EEC	(90, 91)

CC	miR-145	↓ Expression p53 dependent in HPV+ CC cells	Suppresses p53 inhibitors and impedes invasion of HPV+ CC cells	Possibility in employment as prognostic marker; decrease linked to aggressiveness and poor prognosis	(92, 93)
	miR-375	↓	Suppresses cell migration and invasion via targeting SP1 in CC cell lines	Marginal trend observed that miR-375 expression is elevated in chemotherapy resistant patient samples, further samples needed; association with drug sensitivity in BC observed as well	(94, 95)
	miR-181b	↑	AC9 is targeted directly by miR-181b, promotes proliferation, and inhibits apoptosis	Further samples needed to verify elevated miR-181b as a marker	(96)
	miR-143	↓	Targets Bcl-2 abrogates tumor development suppresses apotosis	No link to histology found in samples; clinical application researched	(4, 97, 98)
	miR-126	↓	Up-regulation increases sensitivity to bleomycin targets ADM	Found in serum, correlated with FIGO stage, histological grade, lymphatic invasion, distant metastasis; miR-targeting therapeutic under investigation	(4, 99–101)
	Let-7 family	↓	Targets HAS2 linked to cell survival, invasion	In comparison to normal tissue, cell lines displayed let-7b/c ↓; validation on tumor samples needed	(102, 103)
	miR-100	↓	Targets PLK1 protein	In high-grade cervical lesions and CC, miR-100-PLK1 axis not as distinct, hence this correlation may occur relatively late in cervical tumorigenesis	(104)
	miR-20a	↑	Cell proliferation, migration, and invasion targets TNKS2	Lymph node metastasis, histological grade and tumor diameter	(105, 106)
	miR-21	↑	Targets CCL20, PDCD4 stimulation of cell growth	High in serum of CC patients; miR-targeting therapeutic under investigation	(3, 4)
	miR-29, 29a	↓	Targets HSP47, increases p53 protein level when transfected into HeLa cells, inhibits migration and invasion	miR-29 is connected to HPV infection, further research necessary	(107–109)
	miR-200a	↓	Impacts regulation of cell adhesion	Based on miR-200a and miR-9, two groups with significantly different overall survival rates could be established; indication for miR-200a delivery in patients	(110)

*BC, breast cancer; BCSC, BC stem cells; OC, ovarian cancer; EAC, endometrioid adenocarcinoma; EEC, endometrioid endometrial carcinoma; HPV+, human papilloma virus positive; diff, differentiated; n/a, not available*.

Although multiple research groups have studied diet and dietary patterns in correlation to female cancers, many lack investigations on the miR level. In 2014, a study on the Mediterranean diet versus the Western diet and their implications on BC risk was conducted. It implied that the consumption of vegetables and fruits has a protective effect on TNBC cancer patients. Deciphering the link between diet patterns and tumors of HER2-status, the study was the first to report that the Mediterranean diet had a strong protective impact on TNBC. Furthermore, the positive correlation between a Western dietary pattern and BC risk in general, especially in premenopausal women, was implied (111). Along with these findings, McCann et al. discovered that the dietary intake of lignans is associated with clinical BC characteristics. Lignans are plant-derived polyphenolic substances and available in flaxseeds, nuts, fruits, vegetables, and other foods (112). Another striking finding of our group toward a possible future therapy was the reactivation of ERα in the TNBC cell line MDA-MB-231 through the soy isoflavone genistein, especially in co-treatment with trichostatin A. Molecular mechanisms, specifically on the miR basis, are under current investigation (113). Consequently, finding a way to stimulate miRs with a beneficial effect on the outcome of a patient through adjusting the patient’s diet would be valuable and convenient.

### Aberrant miR profiles in gynecological cancers – an overview

#### Ovarian Cancer

There are three general subgroups of ovarian cancer (OC), germ cell, stromal, and epithelial tumors, with the latter being the most prominent. A common feature of OC is a disease relapse within 2 years (114). Combinational treatments with dietary compounds like epigallocatechin gallate (EGCG) from green tea and sulforaphane from cruciferous vegetables targeting resistant cells seem promising, and the evoked effects by those kinds of treatments need to be further elucidated on the miR level (18, 115).

Especially in terms of OC with its unspecific symptoms, reliable prognostic and predictive biomarkers are avidly sought for. While prognostic markers specify the likely outcome of a disease when the patient is untreated, predictive biomarkers help to determine the patient who will most likely respond and thus benefit from the therapy (116). Being able to differentiate between endometriosis and endometrioid cancer based on the miR profile is a step closer to prognostic markers. Identifying miR signatures that shed light on which patient will respond to anti-angiogenesis therapy in addition to classic therapy is an important goal to identifying predictive biomarkers. Putative biomarkers for epithelial ovarian cancer (EOC) in the blood may be, i.e., miR-205/let-7f and miR-30-1; however, these miRs need further verifications for their application (10).

Similar to BC, where the tumorsuppressor miR-497 has been reported to target cyclin E1, and low BRCA1 expression augmented the incidence risk, miR-479 and BRCA1 are altered in OC as well. In OC cell lines (OVCAR-3, SK-OV-3, HO-8910, HO-8910PM) as well as serous cystadenoma specimens, a decline in miR-497 expression was observed. Patients with serous cystadenoma were shown to have a correlation between miR-497 down-regulation, higher expression of the pro-metastatic factor SMURF1, and shorter overall survival. This suggests that restoration of miR-497 levels may impede OC metastasis (48, 49).

While miR-9 has been associated with tumor cell motility and metastasis in BC, its function as tumorsuppressor-miR through inhibiting the talin1/FAK/AKT pathway has been described in OC (49). miR-9 was found to sensitize ovarian xenograft tumors to cisplatin and PARP inhibitors, and its overexpression led to a higher apoptosis index in xenograft tumor sections (48). An inverse correlation between BRCA1 and miR-9 expression linked to a platinum resistance was stated, and with BRCA1 being critical in the cellular damage response; miR-9 up-regulating compounds are of great interest for OC (39).

When it comes to dealing with different miR signatures, there are not only varying patterns between EOC subtypes but also between primary tumors and their metastases. A study by Vaksman et al. was the first to prove that miR signatures are variable depending on the tumor progression status. They characterized three different sets of miRs that were highly expressed; the first one was overlapping in primary carcinomas and effusions, the second was overexpressed in primary carcinomas, and the last in effusions (117).

#### Uterine and Cervical Cancer

Uterine cancer (UC) is subdivided into endometrial cancer and uterine sarcoma. Endometrial cancer is rather common among Caucasian women, whereas African American women have a higher risk of fatality due to endometrial cancer (85, 118). In formalin-fixed paraffin-embedded (FFPE) endometrial adenocarcinoma tissues, differential expression of miRs between normal and malignant tissue was investigated by Jurcevic et al. In this study, 138 miRs with a difference in the expression pattern between normal and malignant tissue were found. In consensus with findings of the female cancer types aforementioned, a discrimination of early and advanced endometrial tumors was possible on the basis of miR profiles. miR-181b, for instance, was first reported as increased in endometrial adenocarcinoma, targets TIMP3 and tissue inhibitor metalloproteinase-3, and belonged to the group of the 138 miRs. Along with miR-181b, miR-148b and miR-355, which in turn target genes that play a role in the Wnt signaling pathway, were proven to have a significant higher expression in the tumor samples (85).

Most cervical cancers (CC) are squamous cell carcinomas and begin in the majority of cases in the transformation zone, mostly caused by human papilloma virus (HPV) (119). Although immunizations for HPV and the availability of Pap and HPV testing are useful, the knowledge with respect to whether a primary lesion leads to squamous cell carcinoma would influence treatment and avoid unnecessary concerns of the patient. miR-145 could be one possible marker. Wang et al. found this miR to be significantly down-regulated in CC compared to the control; patients with late International Federation of Gynecology and Obstetrics (FIGO) stage CC in comparison to those with early FIGO stage showed a significant decrease as well. Even when correlating lymph node metastasis-positive patients with lymph node metastasis-negative patients, or patients with vascular invasion/HPV infection, a significantly lower level of miR-145 could be observed. Regarding the overall survival time, Kaplan–Meier analysis revealed a proportional relation between miR-145 down-regulation and short overall survival time. Hence, miR-145 may serve as a prognostic factor in the future (93). Similarly, Yang et al. published that a down-regulation of miR-126 leads to a poor prognosis in patients with CC (100). It was demonstrated that the occurrence of host miRs attributed to viral oncoprotein E6 or E7 made it possible to distinguish between normal cervix from cervical intraepithelial neoplasia and CC by calculating the ratio of miR-25/92a and miR-22/29a (120).

The emerging role for miRs as gene network regulators may facilitate the classification of different tumor phenotypes revealing new possible targets and therapies for patients. Table 1 lists examples of miRs and their indications as well as whether these exemplary miRs can be employed as biomarkers. An extensive overview of the miR abundance and regulation, respectively, cataloging and categorizing tumor types is an important goal approaching a ubiquitous application of miRs in individualized medicine. The determination of how diet can protect and prevent not only occurrence but recurrence of female cancers through altering the epigenetic modulator miR is one crucial aspect in the fight against female cancers, and will be reviewed in the following section.

## Phytochemicals, Their Implications, and Impact on miR Levels in Female Cancers

As measured by the publications of articles and books, the awareness of the importance of a balanced nutrition emerges worldwide. Yet, there is comparatively little known about the impact of compounds contained in a healthy diet on miR profiles. Many of those compounds have anticancer as well as other beneficial impacts such as anti-inflammatory, anti-microbial, or anti-oxidative that are empowered to reduce mortality (2, 121). With sulforaphane, EGCG, genistein, resveratrol, and curcumin among the most common compounds studied, there are decisive approaches toward the question of what is healthy, and in which combination the best synergistical effects can be achieved. The search for new potential antitumor drugs as well as the further elucidation of novel anti-tumor compounds such as brusatol, thymoquinone, or methylaervine will be an important part in phytochemical research (122–124).

**Figure 2:**
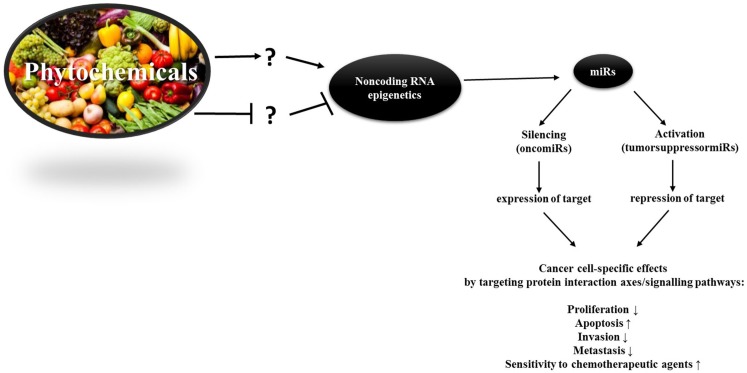
**The idea of the epigenetic diet**. Phytochemicals can alter miR levels and demonstrably up-regulate or silence them. Because oncomiRs and tumorsuppressormiRs are cell-specific, their function in every different cell needs to be ascribed before being able to make consistent statements on the impact of phytochemicals on onco/tumorsuppressormiR-levels. To date, only little is known about the structure-activity relationship of phytochemicals and miR genes. Hence, ? stands for the lack of knowledge concerning the molecular manner in which phytochemicals alter miR levels – whether this occurs indirectly or through direct binding to miRs or miR genes in female cancers.

There is a plethora of phytochemicals, which have an impact on epigenetic processes such as DNA methylation, histone modifications, and non-coding RNA (Figure 2). Because of the frequent occurrence of epigenetic aberrations in cancer, achieving a transition of these through nutrition is a simply available, promising approach to cancer prevention. Not only phytochemicals but also caloric restriction plays key roles in mitigating biological processes that yield cancer (125). Understanding how to alleviate tumorigenic pathways through diet is thus a straightforward approach advantageous for everyone through primary, secondary, and tertiary chemoprevention. Along with other groups we showed, for example, that phytochemicals can enhance effects of chemotherapeutics like Cisplatin, even in chemotherapy-resistant cell lines. Furthermore, we found a verifiable decrease in cell viability through the employment of phytochemicals in a combinatorial manner (115, 126, 127).

Aside from quantity of foods containing chemopreventive compounds, the quality of these foods appear to be effectual as well. Compelling results were generated by Zhang et al. suggesting that horizontal miR transfer from plants to humans is possible. To be precise, miR-168a, abundant in rice, was found to be enriched in the sera of Chinese probands (128). These results make the consumption of genetically modified fruit and vegetables questionable, although the cross-kingdom delivery of diet-derived miRs could not be reproduced (129). Conflating these aspects further underline the interests of investigating dietary compounds. Table 2 reviews a summary of the hereafter presented nutraceuticals and their impact on miR patterns, including signaling pathways in female cancers.

**Table 2:** **Overview of studies indicating effects of phytochemicals on miR levels and cellular reactions as well as pathways in female cancers**.

Phytochemical	Cancer model	miR regulation *↓ ↑*	Effects	Reference
Curcumin	OC SKOV3	miR-9 ↑	Apoptosis by inhibiting activation of AKT and FOXO1	(49)
	BC MCF-7 stimulated with BPA	miR-19a ↓	Modulates PTEN/AKT/p53 axis in favor of stopping proliferation and cell cylce progression	(130)
		miR-19b ↓		
	BC MCF-7, SKBR-3, Bcap-37	miR-15a ↑	Bcl-2 down-regulation	(131)
		miR-16 ↑	Induction of apoptosis	
	BC MDA-MB-231	181b ↑	Down-regulates MMP-1, MMP-3, CXCL1 and -2	(132, 133)
		miR-452-3p ↑	Inhibition of NFκB activation	
		miR-483 ↑	
		miR-423 ↑	
		miR-296 ↑	
		miR-181d ↑	
		miR-498 ↑	
		miR-320 ↑	
		miR-373-3p ↑	
		miR-519e-3p ↑	
		let-7e ↓	
		let-7c ↓	
		miR-503 ↓	

Curcumin in combination with Emodin	BC MDA-MB-231 and MDA-MB-435	miR-34a ↑	Bcl-2 and Bmi-1 down-regulation	(134)
			Inhibiting proliferation, increasing apoptosis	

Genistein	BC MDA-MB-435, Hs578t	miR-155 ↓	Up-regulates PTEN, FOXO3, CK1α, p27	(135)
			Cell growth ↓	
			Apoptosis ↑	
	OC SKOV3	miR-27a ↓	Sprouty2 mRNA and protein ↑	(136)
			Proliferation ↓	
			Migration ↓	
	OC UL-3A	miR-135 ↑	ERα and ERβ ↑	(137)
		miR-765 ↑	Migration ↓	
		miR-122a ↑	
		miR-137 ↑	
		miR-196a ↑	
		miR-204 ↑	
		miR-206 ↑	
		miR-217 ↑	
		miR-331 ↑	
		miR-449b ↑	
		miR-454 ↑	
		miR-501 ↑	
		miR-515 ↑	
		miR-578 ↑	
	OC UL-3B	miR-135 ↑	ERα and ERβ ↑	(137)
		miR-765 ↑	Migration ↓ (lower as in UL-3A)	
		miR-517c ↑		
		miR-7 ↑		

Resveratrol	BC MDA-MB-231-luc-D3H2LN	miR-141 ↑	Inhibits BC invasion and CSC phenotype, resveratrol induces Ago2 expression thus promoting RNAi	(138)
		miR-200c ↑		
		miR-26a ↑		
		miR-34a ↑		
		miR-125a-3p ↑		
		miR-126 ↑		
		miR-128 ↑		
		miR-185 ↑		
		miR-193b ↑		
		miR- 195 ↑		
		miR-196a ↑		
		miR-335 ↑		
		miR-340 ↑		
		miR-497 ↑		
		Putative oncomiRs:		
		miR-378-3p ↑		
		miR-10b ↑		
		miR-132 ↑		
		miR-222 ↑		
	BXC MCF-10A	miR-16 ↑	n/a	(138)
	BC MDA-MB-231-luc-D3H2LN	miR-143 ↑		
	BC MCF-7			
	BC MCF-7ADR			
	BC ACI rats	miR-10a (↑)	Inverse correlation of miR-129, -204, -489, and DNMT3b in normal tissue; in tumor tissue, miR-489 and DNMT3b inversely correlated Resveratrol led to demethylation of *RASSF-1α*	(139)
		miR-10b (↑)		
		miR-21 ↑	
		miR-129 ↑	
		miR-204 ↑	
		miR-489 ↑	
	BC MCF-7	miR-663 ↑	Retardation of cell division	(140)
		miR-744 ↑	eEF1A2 mRNA and protein expression, thus silencing of EEF1A2	

DIM	BC MCF-7	miR-21 ↑	Cell cycle arrest, down-regulation of Cdc25A	(141)
			ER or p53 genotype seem crucial for DIM induced miR-21 ↑	

DIM and Herceptin	BC SKBR3	miR-200a ↑	FoxM1 ↓ pAKT ↓	(142)
		miR-200b ↑	NFκB p65 ↓	
		miR-200c ↑	
	BC MDA-MB-468	miR-200a ↓	Cytotoxicity	(142)
		miR-200b ↓	FoxM1 ↓	
			NFκB p65 ↓	

Sulforaphane	BC MCF10DCIS stem-like cells	miR-140 ↑	Colony/mammosphere formation ↓	(143, 144)
		miR-21 ↑	ALDH1 and expression SOX9 ↓	
		miR-29 ↓	Differetial miR pattern in exosomes	

I3C	MCF-7, MCF10A as control	mir-34a ↑	Cell-cycle arrest, p53 dependent CDK4 suppression	(145)

Polyphenon-60	BC MCF-7	let-7a ↑	Inhibits cell growth	(146)
		miR-107 ↑		
		miR-548m ↑		
		miR-720 ↑		
		miR-1826 ↑		
		miR-1978 ↑		
		miR-1979 ↑		
		let-7c ↓		
		let-7e ↓		
		let-7g ↓		
		miR-21 ↓		
		miR-25 ↓		
		miR-26b ↓		
		miR-27a/b ↓		
		miR-92a ↓		
		miR-125a-5p ↓		
		miR-200b ↓		
		miR-203 ↓		
		miR-342-3p ↓		
		miR-454 ↓		
		miR-1469 ↓		
		miR-1977 ↓		
		Suppresses:		
		miR-30b-3p		
		miR-29a		
		miR-221		
		miR-936		
		miR-1249		
		miR-200a		
		miR-424		

Pomegranate polyphenols	BC BT-474	miR-155 ↓	Cancer cell-specific growth suppression	(147)
	BC MDA-MB-231	miR-27a ↓	SHIP-1 ↑	
	BC BT474 xenografts in nude mice		ZBTB10 ↑	
			Sp1, Sp3, and Sp4 ↓	
			PI3K-dependent pAKT ↓	

Ellagic acid	ACI rat model	miR-122 ↑	ERα ↓	(148)
		miR-127 ↑	Bcl-2 ↓	
		miR-182 ↓	Bcl-w ↓	
		miR-183 ↓	cyclin D1 ↓	
		miR-206 ↑	cyclin G1 ↓	
		miR-375 ↓	FOXO1 ↑	
			FOXO3a ↑	
			RASD1 ↑	

Betulinic acid	MDA-MB-231 xenograft	miR-27a ↓	Abrogation of proliferative, angiogenic phenotype: repression of survivin, Sp 1, 3, 4, VEGF and VEGFR Myt-1 ↑ and thus cell cycle arrest at G_2_/M (pcdc2)	(149, 150)
			ZBTB10 ↑ in lungs of mice β2-microglobulin ↓	
	MDA-MB-231 xenograft in nude mice	miR-106a ↓	ZBTB4 ↑	(150)
		miR-106b ↓	Sp1, Sp3, Sp4 ↓	
		miR-20a ↓	EZH2 ↓	
	BC BT474	miR-27a ↓	Effects of betulinic acid CB1 and CB2 receptor dependent:	(151)
	BC MDA-MB-453; both overexpressing ERBB2		Sp1, Sp3, Sp4 ↓	
			YY1 ↓	
			ERBB2 ↓	
			ZBTB10 ↑	

ACA	CC Ca Ski, CC HeLa	miR-629 ↑	Abates cellular gluthatione levels	(152)
		miR-487a ↑		
		miR-483-3p ↑		
		miR-376a ↑		
		miR-342-3p ↑		
		miR-212 ↑		
		miR-1262 ↓		
		miR-875-3p ↓		
		miR-517 ↓		
		miR-411 ↓		

ACA and Cisplatin	CC Ca Ski, CC HeLa	miR-922 ↑	ACA enhances cisplatin efficacy by preventing its inactivation if administered *before* cisplatin	(152)
		miR-744 ↑		
		miR-523 ↑		
		miR-210 ↑		
		miR-138 ↑		
		miR-1271 ↓		
		miR-224 ↓		
		miR-21 ↓		

Garcinol	BC MDA-MB-231	miR-200b ↑	Induction of apoptosis and MET	(153)
	BC BT-549	miR-200c ↑	NFκB p65 ↓	
		let-7a/e/f ↑	Inhibition of Wnt signaling (nuclearβ-catenin ↓)	
			Vimentin ↓	
			ZEB-1 ↓	
			ZEB-2 ↓	
			E-cadherin ↑	

Glyceollins	BC MDA-MB-231	miR-181 c/d ↑	Apoptosis	(154)
	BC MDA-MB-468;	miR-22 ↑	repression of SLC7A11	
	xenografts	miR-26b ↑		
		miR-29b/c ↑		
		miR-30d ↑		
		miR-34a ↑		
		miR-195 ↑		
		miR-663 ↑		
		miR-193a-5p ↓		
		miR-197 ↓		
		miR-224 ↓		
		miR-486-5p ↓		
		miR-542-5p ↓		

Matrine	BC MCF-7	miR-21 ↓	miR-21/PTEN/AKT axis targeted:	(155)
			pAKT ↓	
			pBAD ↓	
			p21 ↑	
			p27 ↑	

Artemisinin and artesunate	MCF-7, T47D	Mir-34a ↑	Cell-cycle arrest, p53 independent CDK4 suppression	(145)

Ascorbic acid	BXC MCF-10A,	miR-93 ↓	NRF2 and NRF2 related genes ↑	(156)
	BC T47D		MCF-10A: decrease in colony and mammosphere formation	

Calcitriol	OC OVCAR3	miR-498 ↑	hTERT mRNA stability ↓	(157)
	[OC A2780, OC A2780-CP, OC C13]			

Calcifediol	BXC MCF10A	miR-182 ↓	Protection of BXC from oxidative/low serum/hypoxia stress	(158)
	BXC MCF12F		suppression of cell proliferation	

*CSC, cancer stem cell; ↑, marginally significant trend; OC, ovarian cancer; CC, cervical cancer; BC, breast cancer; BXC, non-cancerous breast epithelial cell line; n/a, not available*.

### Curcumin

Curcumin can be obtained from the yellow rhizome of *Curcuma longa* or turmeric and has health promoting virtues resulting in telomerase inhibition, decrease of HDAC 1, 3, and 8 protein levels, as well as induction of DNA hypomethylation. At present, there are 50 studies listed by the NIH for the use of curcumin in cancer patients. Although curcumin has been reported to be safe in high doses (in rats a LD of 5–10 g/kg), its bioavailability even with high dosage is low (159–161). Due to the fact that numerous bioavailability studies in rodents as well as humans yielded plasma levels of curcumin below 1 μmol/L, efforts in therapeutic drug development are being made. Synthesizing either nanoparticles conjugated with curcumin or nanocurcumin itself, curcumin-analogs or creating liposomes is one approach. Another is to add piperidin, an alkaloid of black pepper. It enhances curcumin’s bioavailability by inhibiting its glucuronidation which in turn affects the phase-II-metabolism. Interestingly, curcumin conjugates have been proven to negatively influence their bioavailability through the induction of transcriptional factor Nrf2, accounting for the transcription of multidrug resistance-related proteins which facilitate the active efflux from enterocytes (160, 162). Moreover, in the CC cell line SiHa (HPV16+), curcumin has been shown to be a strong but non-specific inhibitor of phosphorylation at Y705 of STAT3 signal transducer and activator of transcription 3, which in turn can promote oncogenesis when constitutively active (163).

Owing to its broad range of palliative features, curcumin targets various members of cellular signaling pathways. Not only does it play a role in the activation of the ambivalent transcription factor Nrf2 but it also impacts NFκB, AKT, FOXO1, PTEN, p53, and various other members of signaling pathways (49, 130, 159, 160, 164). A curcumin-dependent modulation of the aforementioned pathway mediators can be brought about demonstrably by miRs. In the human Caucasian ovary adenocarcinoma cell line, SKOV3, Zhao et al. found that curcumin treatment dose-dependently resulted in an elevated expression of miR-9, inducing apoptosis by inhibiting AKT activation and FOXO1 phosphorylation at unchanged protein levels. Since Weir et al. were able to show that curcumin induces apoptosis in cisplatin-resistant human OC cells through caspase-3 activation and PARP cleavage, the question of whether miR-9’s pro-apoptotic action was achieved similarly was posed. It was shown that miR-9 activated caspase-3 as well as PARP degradation in SKOV3 cells (49).

**Figure 3:**
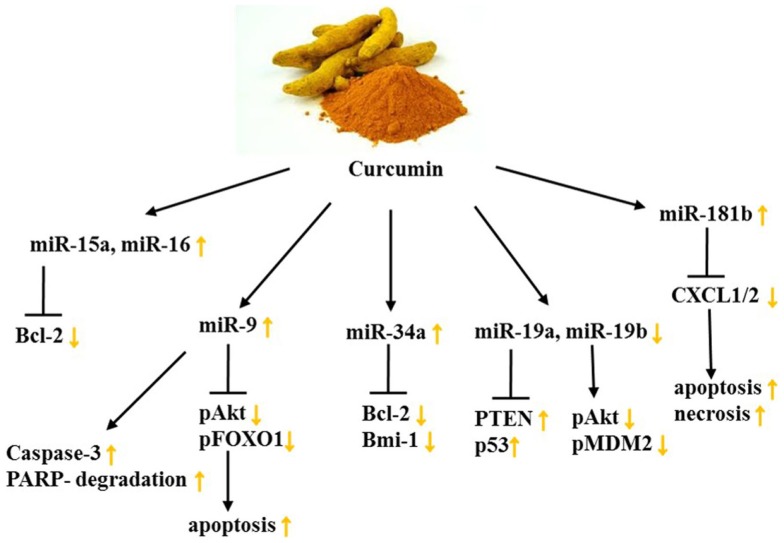
**Curcumin’s impact on miR levels and their targets in female cancers**. Yellow arrows demonstrate effect of curcumin on indicated targets/processes on female cancer models. See text for indicated changes, as each presented axis represents findings illustrated in the text.

A further pathway targeted by curcumin is PTEN/AKT/p53 in BC. This was discovered by a study in 2014 examining the impact of curcumin on BPA on stimulated MCF-7 BC cells. As mentioned earlier, BPA is closely connected to mastocarcinoma because it acceleratedly impairs proliferation and apoptosis pathways. In this study, miR-19, a member of the miR-17-92 cluster, was recognized to be up-regulated upon BPA stimulation, leading to a decreased expression of PTEN and p53 in addition to increased levels of p-AKT, p-MDM2, and PCNA. Curcumin treatment, however, compromised BPA-induced proliferation and cell cycle progression through modulation of miR-19a and miR-19b (Figure 3) (130).

Curcumin acts as an anti-inflammatory agent and because chronic inflammation is a considerable risk factor for metastasis, the impact of curcumin on the miR-dependent regulation of the proinflammatory cytokines CXCL1 and -2 was examined. This was done by assessing miR expression with microarrays in the TNBC cell line MDA-MB-231 as well as in primary ER+, HER2-breast tumor samples. Hereby, 3 miRs were decreased and 10 miRs increased at least 2.5-fold in response to curcumin dosage of 25 μM (Table 2). Among these miRs detected, miR-181b was found to down-regulate CXCL1 and -2 through direct binding of the 3′UTR, as well as MMP-1 and -3, constituting the anti-metastatic effect of curcumin. Moreover, the group could attribute the anti-proliferative, pro-apoptotic, and pro-necrotic traits of curcumin to the curcumin-induced up-regulation of miR-181b as well as the inhibition of NFκB in BC cells (132, 133).

Bcl-2 is a key oncogene since it can alter mitochondrial permeability and cytochrome c release, thus it can favor cell survival. Due to the fact that it is overexpressed in numerous cancer types, including female cancers, finding phytochemicals which target Bcl-2 is advantageous. Yang et al. depicted a curcumin-dependent apoptosis in MCF-7 cells through down-regulation of Bcl-2 effectuated by increased miR-15a and miR-16 levels. Other BC cell lines, namely SKBR-3 and Bcap-37, showed similar results after the administration of 60 μmol/L curcumin (131). Because miR-34a’s tumorsuppressive character had been previously described, Guo et al. studied if curcumin impedes cancer cell growth and metastasis through miR-34a. In combination with emodin, a phytochemical derived from *Rheum palmatum* (rhubarb root) that has broadly found application in Chinese medicine, the effect of curcumin was enhanced. Not only did a combinatorial treatment of curcumin and emodin inhibit TNBC cell proliferation, increase apoptosis, and suppress invasive potential but also synergistically incremented miR-34a expression. As a result, Bcl-2 and Bmi-1 showed decreased levels in MDA-MB-231 and MDA-MB-435 cell lines (134). Investigating synergisms of curcumin and Cisplatin as well as EGCG and Cisplatin, Yunos et al. could furthermore demonstrate in the human ovarian tumor models: A2780, A2780^cisR^, and A2780^ZD0473R^, growth inhibitory effects, as well as a potentiating outcome of the treatments. Changes on the miR-level, however, remain unknown (165).

Moreover, curcumin has been proven to alter miR profiles in various other cancer types such as pancreatic, prostate, colorectal, and bladder cancer to name a few. Interestingly, several of the currently described miRs targeted by curcumin in these cancers are known to be dysregulated in female cancers as well. One prominent example is the let-7 family and its down-regulation in a diverse range of cancer types (166, 167). Ali et al. demonstrated that in the human pancreatic cancer cell lines COLO-357, MIAPaCa-2, and BxPC-3, the treatment with a curcumin analog led to a re-expression of let-7 along with miR-143 and a decrease of miR-21. This triggered the inhibition of cancer cell growth *in vitro* and *in vivo*. A corroboration of whether curcumin has a similar impact on miR-21, 143, and let-7 in female cancers may be important, as miR-21 overexpression is associated with invasive CC, miR-143 overexpression was linked to Bcl-2 inhibition in HeLa cells and restoration of let-7b in OC cells dramatically reduced tumor cell growth (98, 167, 168). Since Zhang et al. suggest a worse prognosis of endometrioid cancer patients with a down-regulation of miR-143, findings of whether curcumin induces the expression of miR-143 in endometrioid carcinomas like in pancreatic cancer cell lines may be of special interest (169).

In the context of nutrition, BC treatment, and chemotherapy, a striking discovery has been made. Somasundaram et al. observed antagonistic effects between curcumin and chemotherapeutic agents in MCF-7, MDA-MB-231, and BT-474 human BC cells, with an inhibition by up to 70%. This was verified in an *in vivo* model of human BC in mice. Since curcumin is able to inhibit the formation of reactive oxygen species (ROS) and the c-Jun NH_2_-terminal kinase (JNK) pathway, the group suggested that patients receiving camptothecin, mechlorethamine, doxorubicin, or other chemotherapeutics, which exert their anti-tumor effects by generating ROS and activating JNK, ought to take precautions when consuming curcumin (170). The manner of how miRs exert their function in this case, however, is not known and might be a missing link toward grasping the molecular mechanisms of this counteraction more profoundly.

In conclusion, curcumin or its analogs are capable of altering miR signatures in female cancers (Figure 3; Table 2). The studies suggest that curcumin-dependent changes in the miR profile modulate cell growth and induction of apoptosis as well as necrosis and invasive traits of female cancers. However, these changes are heavily cell context-dependent, and further research on which patient will benefit from curcumin consumption and which rather ought to avoid it is of particular necessity. Implications for curcumin on the effect of miR patterns in female cancers, its synergisms with chemotherapeutics as well as increasing its bioavailability sustainably will need further investigation in the future.

### Genistein

The consumption of soy products has gained significance due in part to epidemiological studies indicating increased breast and prostate incidences in the Western world in contrast to Asia, where a soy-based diet is typically consumed. Genistein, a predominant soy isoflavone, is known to impact cancer cell proliferation, angiogenesis, induction of differentiation by directly targeting molecular signaling pathways like NFκB and AKT, as well as modulating epigenetic events, especially DNA methylation. While curcumin has low bioavailability, this is not the case for genistein: a soy-rich diet results in a noticeable plasma level of genistein (2, 159, 171).

In prostate cancer, a mix of isoflavones containing 70.5% genistein were shown to demethylate promoters of miRs acting against tumor invasion and proliferation (e.g., miR-29a and miR-1256). However, in female cancers, genistein holds an ambivalent role, specifically in BC (172, 173). On one hand, as our group already reported, genistein significantly inhibits early breast tumorigenesis in a dose-dependent manner by increasing the expression of tumorsuppressor genes like *P21* and *P16* and decreasing the expression of BMI1 and c-MYC through promoter alterations (174). On the other hand, genistein stimulated tumor growth at low concentrations and extenuated the effect of tamoxifen due to its phytoestrogen features in ER+ BC (173). Hence, the finding of an adequate exposure window is critical and BC risk may be reduced by a diet high in soy during childhood and adolescence. In adult women as well as during pregnancy, beneficial genistein consumption has been questioned. Nevertheless, genistein induced the inhibition of human telomerase reverse transcriptase (hTERT) and three DNMTs in BC cell lines (175).

Probably due to its pleiotropic effects, little information on the effect of genistein on miR levels in BC has been published. In MDA-MB-435 and Hs578t BC cells, however, genistein was found to inhibit the expression oncomiR-155 while up-regulating its targets such as PTEN, FOXO3, CK1α, and p27 at low physiological concentrations (135). Notably, in the five subgroups of BC, a differential expression between ER− and ER+ tumors of miR-155 prevails (18).

Aside from the limited findings with respect to genistein altered miR levels in BC, Xu et al. reported an overexpression of miR-27a in 20 FFPE ovarian tissue samples. This overexpression was abrogated upon 50 μM genistein treatment in SKOV3 OC cells, followed by a significant increase in mRNA and protein level of the putative target of miR-27a, Sprouty2. Sprouty2, an intracellular regulator of receptor tyrosine kinase signaling associated in processes like cell growth and differentiation, is a target gene of the Wnt/β-catenin pathway. Interestingly, Sprouty2 is up-regulated in the majority of colon carcinomas, while an increase in the SKOV3 cell line seems to be favorable (136, 176). Genistein has also been reported to target miR-27a, which in turn affects *ZBTB10* expression in uveal melanoma, thereby raising the question of whether this applies to female cancers as well (177). In MCF-7 and OC cell lines, miR-27a was shown to play a role in cellular processes and signaling pathways. In the former cell line, miR-27a indirectly regulates ERα expression and hormone responsiveness, while in latter it is connected to the ZBTB10-specificity protein pathway (178, 179). Further investigation will be required to determine if genistein exerts its function in female cancer in a similar manner as it does in uveal melanoma.

**Figure 4:**
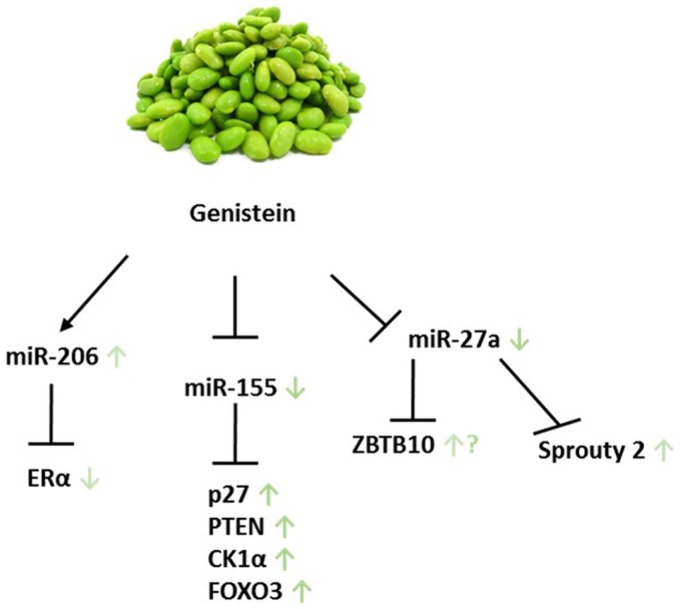
**Genistein and its miR-related targets in female cancers**. Green arrows show according impact on the particular target in female cancer models. ? addresses the following: genistein was shown to target ZBTB10 in uveal melanoma; however, it remains unclear whether this applies to female cancers as well. Displayed axes represent the findings of presented studies in the text.

It has been reported that genistein significantly reduces invasive traits and modifies miR profiles in cell lines derived from a patient with papillary serous adenocarcinoma of the ovary. Compared to the cell line UL-3A, which was collected and cultured at diagnosis, UL-3B cells were isolated after 6 months of failed treatment with Cisplatin/Paclitaxel. Differential expression of miRs after genistein treatment was observed in both cell lines: miR-135 and miR-765 levels were increased (Figure 4; Table 2). UL-3A showed 12 up-regulated miR species: miR-122a, miR-137, miR-196a, miR-204, miR-206, miR-217, miR-331, miR-449b, miR-454, miR-501, miR-515, and miR-578. UL-3B cells, however, only showed an induction in miR-517c and miR-7, indicating that ineffective Cisplatin/Paclitaxel treatment yields considerably less genistein-induced miR profile changes. Western immunoblotting as well as real-time PCR analysis furthermore demonstrated an increase in ERα and ERβ. Interestingly, miR-206, which was up-regulated in UL-3A cells upon genistein treatment, represses ERα in BC cell lines. Because the ratio of ERα/ERβ is high in OC, the group suggests a protective role for ERβ, consequently emphasizing the importance of ERβ-inducing compounds (137).

Isoflavones, genistein in particular, were indicated to have positive effects on endometrial carcinogenesis in a mouse model as well as TNBC (180, 181). Genistein was shown to facilitate the expression of pro-apoptotic factors like PTEN, FOXO3, and p27 through miRs in BC. Due to its structural resemblance to hormones, genistein functions as a phytoestrogen and is able to bind to ERs. It interacts with higher affinity to ERβ than estrogens (137, 182). These results are in favor of genistein application in cancer patients; nonetheless, further studies examining the conditions which render genistein intake as beneficial are needed. In addition, the exposure timing of genistein to female patients seems to be critical. For this, studying genistein-dependent changes in miR profiles opens exciting new avenues.

### Resveratrol

Resveratrol (trans-3, 4′, 5-trihydroxystilbene) is a natural bioactive polyphenol found in red grapes, peanuts, and blueberries for instance. It exhibits a plethora of physiological properties such as anti-oxidant, anti-inflammatory, and anti-cancer by altering cell signaling such as up-regulating the expression of Bax, PUMA, Bim, p53 and down-regulating Bcl-2, Bcl-XL, as well as survivin. Furthermore, resveratrol can cause cell cycle arrest at G_1_ and G_1_/S phases through the induction of the expression of CDK inhibitors leading to growth inhibition accompanied by apoptosis. In MCF-7 BC cells, the modulation of phosphorylated AKT and caspase 9 has been suggested to act as apoptosis trigger. Excitingly, it can also prevent epigenetic silencing of BRCA-1 in MCF-7 BC cells. Whether resveratrol acts as an ER agonist or antagonist appears to be dependent, at least in part, on cell type and dosage of resveratrol (159, 183–187).

**Figure 5:**
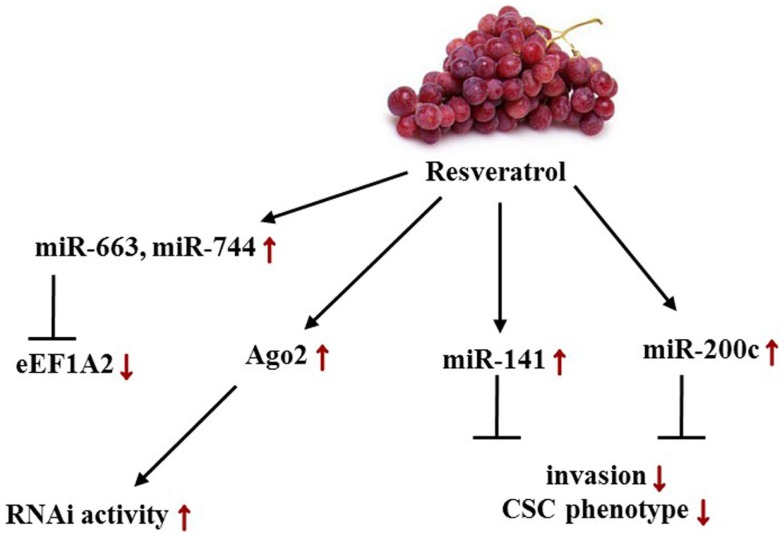
**Effects of resveratrol-induced changes in female cancers on miR levels and its targets/phenotypes/pathways**. Red arrows depict resveratrol-dependent alterations in female cancer models. Displayed axes represent the findings of presented studies in the text.

Given the number of clinical studies listed^2^ , resveratrol is employed frequently for the treatment colon cancer, whereas among female-related diseases, currently only studies for patients with polycystic ovary syndrome are conducted. *In vivo* experiments, however, indicated a clinical significance for resveratrol administration in female cancers (Figure 5). Hagiwara et al. were not only able to show that intraperitoneal injection of 25 mg/kg/day into SCID outbread mice significantly suppressed MDA-MB-231-luc-D3H2LN tumor cell growth but also that CD44+/CD24− cancer stem cells (CSCs) significantly decreased by sixfold upon resveratrol treatment. Hypothesizing that these properties were brought about by a change in the miR level, the group showed convincingly a resveratrol-mediated up-regulation of miR-141 and miR-200c, which are known to be capable of inhibiting BC invasion as well as the CSC phenotype. Irrespective of the miRs aforementioned, an up-regulation of the tumorsuppressive miRs 26a, 34a, 125a-3p, 126, 128, 185, 193b, 195, 196a, 335, 340, and 497 was detected. From the group classified as oncomiRs, levels of 378-3p, 10b, 132, and 222 were increased more than twofold. In MCF10A control cells, a significant up-regulation of primary, mature miR-16 and miR-143 at a concentration of 25 μM resveratrol was observed. Aside from these findings, the group also presented a significant transcriptional induction of Argonaute 2 (Ago2) expression, which in turn enhanced the RNAi activity (138).

The assessment of resveratrol induced effects in estrogen-dependent mammary carcinoma tissue versus normal tissues of August Copenhagen Irish (ACI) rats, which develop BC when exposed to 17-β Estradiol, further underlined the epigenetic impact of this compound. First, resveratrol treatment resulted in a lag of tumor development as well as a significant DNMT3b down-regulation in tumors compared to normal mammary tissue. Second, miR-10a and miR-10b showed a marginally significant increase in tumor tissue related to a low dose treatment of 5 mg/kg/day, while high dose resveratrol treatment of 25 mg/kg/day augmented the expression of miR-21, miR-129, miR-204, and miR-489 more than twofold in tumor tissue. A general trend showed inverse proportional miR levels in tumor tissue compared to normal tissue. Furthermore, an inverse correlation of RNA levels between miR-129, 204, 489, and DNMT3b was seen in normal tissue; in tumor tissue, this was only observed for miR-489. DNMT3b is perceived as the prevalent methyltransferase in breast carcinogenesis; hence, the inverse correlation between DNMT3b and miR-129 and 204 needs further examination with respect to its role in tumor development. As an antipode, resveratrol was moreover found to increase miR-21 expression in the ACI rat model, although miR-21 overexpression correlates with advanced BC stages and is linked to aggressiveness along with hormone insensitivity in HER2+ tumors and to various other female malignancies. Due to the fact that the mammary tumors in this study were hormone sensitive and HER2− and miR-21 might have different implications, further analysis will be needed in this model (139).

In 2002, Anand et al. identified eukaryotic translation elongation factor 1A2 (eEF1A2) as a proto-oncogene in OC. Embodying a key role in protein synthesis by binding aminoacyl-tRNA and transmitting it to the ribosomal A-site, eEF1A2 was found to acquiesce anchorage-independent growth and increase growth of ES-2 ovarian carcinoma cells in nude mice. Recently, a study indicated that miR-663 and miR-744 directly target EEF1A2 expression at mRNA and protein levels. Resveratrol was found to post-transcriptionally down-regulate both mRNA and protein levels by stimulating miR-663 and 744 expression in MCF-7 BC cells implying that it reactivates a miR-mediated silencing mechanism of eEF1A2 (140, 188).

Along with caloric restriction and blueberry powder, resveratrol has demonstrated to have anti-cancer properties (181, 189). Up-regulating miR-663 in THP-1 monocytic cells, impairing prostate cancer cell growth through inhibition of the miR-21/AKT axis as well as decreasing oncomiRs such as miR-7, miR-20b, and miR-1260 in prostate cancer make resveratrol a promising phytochemical in epigenetic cancer chemoprevention (190–192). Because oral application of resveratrol results in 20% bioavailability, one may consider the more stable pterostilbene, with similar indications, including the increase of tumorsuppressor miRs like miR-143 and 200c in MDA-MB-231-luc-D3H2LN cells as a potent alternative (138). Eminently in reference to BC chemoprevention, resveratrol was capable of reducing the incidence and multiplicity by 45 and 55%, respectively (139). Although there have been studies conducted toward its beneficial impact in ovarian, uterine, and CC, the resveratrol induced miR alterations in these gynecological cancer types still remain unknown.

### Phytochemicals contained in cruciferous vegetables

In conjunction with curcumin, genistein, and resveratrol, there have been several discoveries on the impact of phytochemicals, such as 3, 3′-diindolylmethane (DIM), sulforaphane, Indole-3-carbinol, and a plethora of other biologically active components on miR signatures in female cancers. To begin with, DIM, a *brassica* vegetable-derived glucosinolate and a condensation product of idole-3-carbinol, represses growth of xenografted human breast carcinoma cells (MDA-MB-468 or MCF-7) in Balb/c athymic (nu/nu) mice. Regardless of p53 rank and hormone sensitivity, it furthermore induces cell-cycle arrest (at G_1_ and G_1_/G_2_M phase) in both cell lines through impacting cell-cycle regulators, for instance up-regulating p21 levels, and down-regulating Cdc25A. miR-21 on the other hand targets oncogene Cdc25A directly through a binding site in the 3′UTR of Cdc25A. Certainly, this contributes to the not yet fully understood controversial traits of miR-21. DIM treatment resulted in an up-regulation of miR-21 in MCF-7 cells but not in the TNBC MDA-MB-468 p53 mutants, indicating that ER or p53 status is crucial for the DIM-related effect on miR-21 in BC (141, 142).

A major obstacle for HER2+ patients is Herceptin resistance, regardless if gained primarily or secondarily. Circumventing this resistance with the aid of dietary components was addressed in a further study on DIM. Here, a synergism between DIM and Herceptin (Trastuzumab) was ascertained. Combinatorial treatment of SKBR3 cells not only led to the decrease of FoxM1 and phosphorylated AKT, but to a significant increase in miR-200a, miR-200b, and miR-200c as well. Aside from this cell line derived from pleural effusions of breast adenocarcinoma overexpressing Her2/neu, the group employed the TNBC cell line MDA-MB-468. Cotreatment of MDA-MB-468 cells showed cytotoxic effects and a significant up-regulation of miR-200a and miR-200b. Transfection studies in both cell lines revealed that pre-miR-200 in combination with DIM and Herceptin treatment decreased FoxM1 and NFκB p65 expression, indicating that this down-regulation is mediated through miR-200. Hence, important findings toward vanquishing Herceptin resistance as well as providing a new basis for the future treatment of patients with TNBC have been made. Comprehensive investigations on how the synergism of DIM with Herceptin or other drugs such as Paclitaxel works on a molecular basis and applying those findings to cancer therapy will need to be further pursued (142, 193).

**Figure 6:**
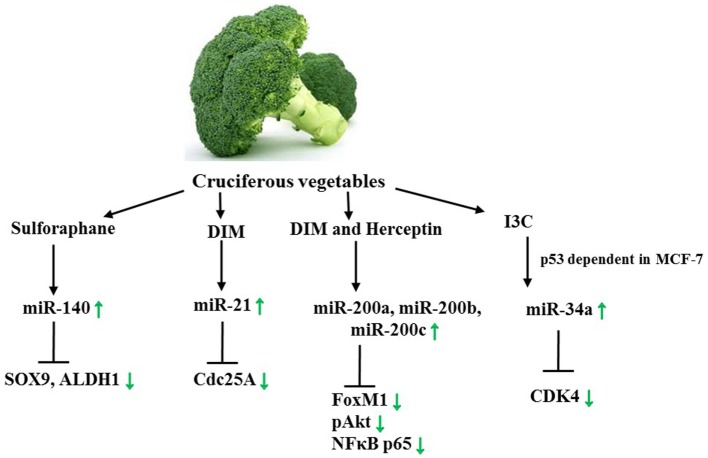
**Axes targeted by Sulforaphane, DIM, and Herceptin as well as I3C**. Green arrows demonstrate specific effect of sulforaphane/DIM in female cancer models. See text for indicated changes, as each presented axis represents findings illustrated in the text.

Another phytochemical obtained from cruciferous vegetables is the isothiocyanate sulforaphane, which has been reported to reduce the risk of cancer development. A plethora of studies on its molecular impacts, including epigenetic effects, have been made, and cues that early-life consumption of sulforaphane as well as exposure of the embryo *in utero* are beneficial have been provided (2). In ductal carcinoma *in situ* (DCIS) stem-like subpopulations, a declined colony/mammosphere formation and ALDH1 expression in addition to differential miR expression in DCIS exosomes were observed upon sulforaphane treatment. To be precise, a higher abundance in miR-140 and a lower abundance in miR-21 and miR-29 were detected in the exosomes. Interestingly, treatment of MCF10A and MCF10DCIS cells revealed that stem cells of the former cell line released more miR-140 than MCF10A non-stem cells, whereas MCF10DCIS stem cells secreted a lower number of miR-140. Sulforaphane, in turn, was suggested to alter the microenvironment by increasing the exosomal miR-140 secretion, thereby affecting cell signaling. miR-140 not only regulates CSCs in luminal subtype invasive ductal carcinoma but also directly targets SOX9 and ALDH1, critical stem cell factors. *In vitro* and *in vivo*, sulforaphane restored miR-140 expression and decreased the tumor volume. These findings provide evidence that the consumption of sulforaphane may eliminate stem cell like cells in DCIS lesions through miR regulation. Indeed, the question whether miR excretion plays a significant role for *in vivo* signaling requires further analysis. Generally, two different assumptions regarding the function of extracellular miRs are being made, either that these miRs are simply byproducts or that they function as intercellular communicators (143, 144, 194). In conclusion, nutraceuticals contained in *brassica* vegetables have been proven to alter the miR expression profile of female cancer (Figure 6) while their impact on miR levels of gynecological cancers is still unexplored.

A very recent finding indicates that Indole-3-carbinol (I3C) is able to induce cell cycle arrest and stimulate miR-34a expression in the BC cell line MCF-7 in a wild-type p53 dependent response. As a result, miR-34a target CDK4 is suppressed. Interestingly, this is one of the first studies aiming at deeper mechanistic insights on the mode of action of phytochemicals and their impact on miR levels in female cancers (145). Thus, further research, especially on how phytochemicals bring about changes in miR levels on a molecular basis is necessary. For this, questions such as in which manner do phytochemicals promote or suppress miR genes ought to be posed.

### Polyphenon-60

Among the most consumed beverages worldwide is tea, and the catechins occurring in green tea are known to be biologically active. We demonstrated that EGCG, a major polyphenol of green tea, induces apoptosis in Paclitaxel- and Cisplatin-resistant OC cells through modulating cellular signals and enzymes like hTERT. The role of miR epigenetics, however, remains to be fully explored (115, 126). Polyphenon-60 (P60), a green tea extract with 60% catechins, inhibits cell growth and influences the miR signatures of MCF-7 BC cells. While 7 miRs, miR-30b-3p, miR-29a, miR-221, miR-936, miR-1249, miR-200a, and miR-424 were found in untreated samples compared to the treated ones, 23 miRs were detected to be differentially expressed upon treatment with 10 μg/ml P60 for 48 h; Seven of these were found increased (let-7a, miR-107, miR-548m, miR-720, miR-1826, miR-1978, and miR-1979); 16 miRs were observed to be decreased (let-7c, let-7e, let-7g, miR-21, miR-25, miR-26b, miR-27a/b, miR-92a, miR-125a-5p, miR-200b, miR-203, miR-342-3p, miR-454, miR-1469, and -1977) (146). Aside from let-7a (down-regulated in breast and OC) and miR-107 (specific to ERBB2 status in BC, found in OC-derived exosomes), the remaining up-regulated miRs have not been detected frequently in the context of female cancer (195–197)^1^. let-7a is down-regulated not only in BC but in cervical and OC as well. The fact that let-7a was among the most highly increased miRs due to treatment is not only demonstrating P60’s anti-cancer potential in BC but poses the question of whether this applies to cervical and OC as well. Whether P60 treatment is beneficial for bladder cancer and various other cancer types along with Alzheimer’s disease or Creutzfeldt-Jakob disease needs further investigation, since reported dysregulated miRs in these medical conditions were altered by P60 (146).

### Pomegranate polyphenols

**Figure 7:**
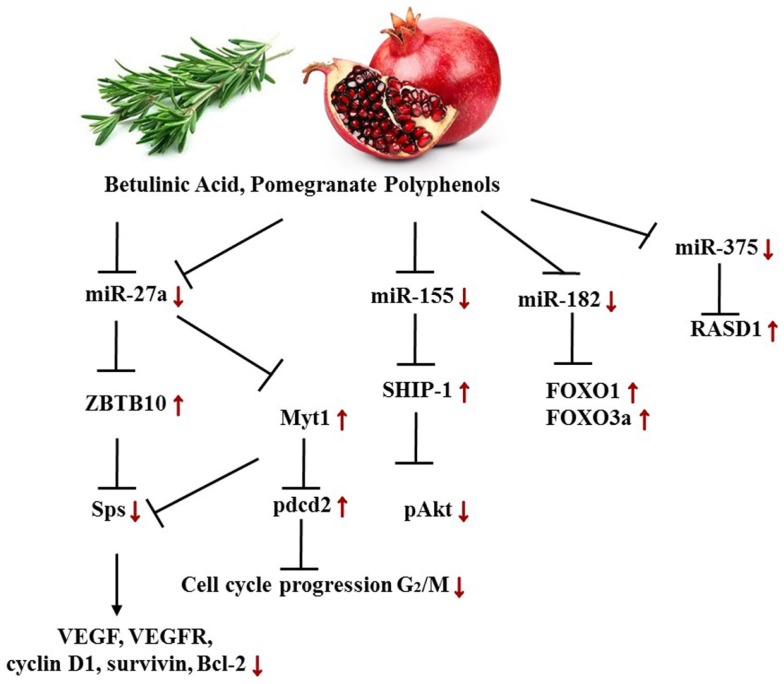
**Targeted axes in female cancer models by pomegranate phenols and betulinic acid**. Displayed axes represent the findings of presented studies in the text.

Pomegranate contains the polyphenols punicalagin A and B (ellagitannins) and delphinidin 3-glucoside, cyanidin-3-glucoside (anthocyanins), as well as ellagic acid glucoside and free ellagic acid. These polyphenols abolish cancer-promoting characteristics of estrogen and sensitize ERα+, tamoxifen-sensitive and, -resistant cancer cells to tamoxifen treatment. Pomegranate extract induces a decrease of miR-155 followed by a rise in SHIP-1 (Inositol 5‘phosphatase) mRNA and protein expression along with an inhibition of the PI3K dependent AKT phosphorylation. The transcription factors Sps (specificity proteins) are frequently overexpressed in a variety of tumors, and can be inhibited by the zinc finger protein ZBTB10. Pomegranate polyphenols were shown to down-regulate miR-27a, a suppressor of ZBTB10, hence leading to an up-regulation of ZBTB10 and an abolishment of Sp (Figure 7). These findings explain to an extent the anti-inflammatory and anti-cancer effects of pomegranate extracts on the molecular basis (147, 198). Thus, disrupting the miR-155-SHIP1 as well as the miR-27a-ZBTB10-Sp axis in human BC by pomegranate or other phytochemicals such as curcumin or betulinic acid appears promising. Munagala et al. moreover demonstrated in an ACI rat model that 3 weeks after primary 17β-Estradiol exposure, the miR profiles changed, and ellagic acid, found in pomegranate and raspberries, abrogated carcinogenesis by repealing miR-122, miR-127, miR-182, miR-183, miR-206, and miR-375 deregulation. As a result, the target proteins, including ERα, Bcl-2, cyclin D1, and cyclin G1, were decreased in either mRNA or protein expression levels or both respectively. The FOXO proteins FOXO1 and FOXO3a, in contrast, increased upon ellagic acid treatment, ascribed to miR-182 down-regulation. Similarly, miR-375 expression was diminished due to the treatment and thus led to a higher abundancy in RASD1 protein (148).

## Other Phytochemicals and Nutraceuticals

Aside from the prominent phytochemicals such as curcumin, genistein, or resveratrol, further remarkable compounds found in nature have been reported to affect miR signatures in female cancers. This section addresses betulinic acid, 1′S-1′-acetoxychavicol acetate, garcinol, glyceollins, matrine, artemisinin, and certain vitamins with respect to miR levels and the resulting effects in female cancer models.

### Betulinic acid

**Figure 8:**
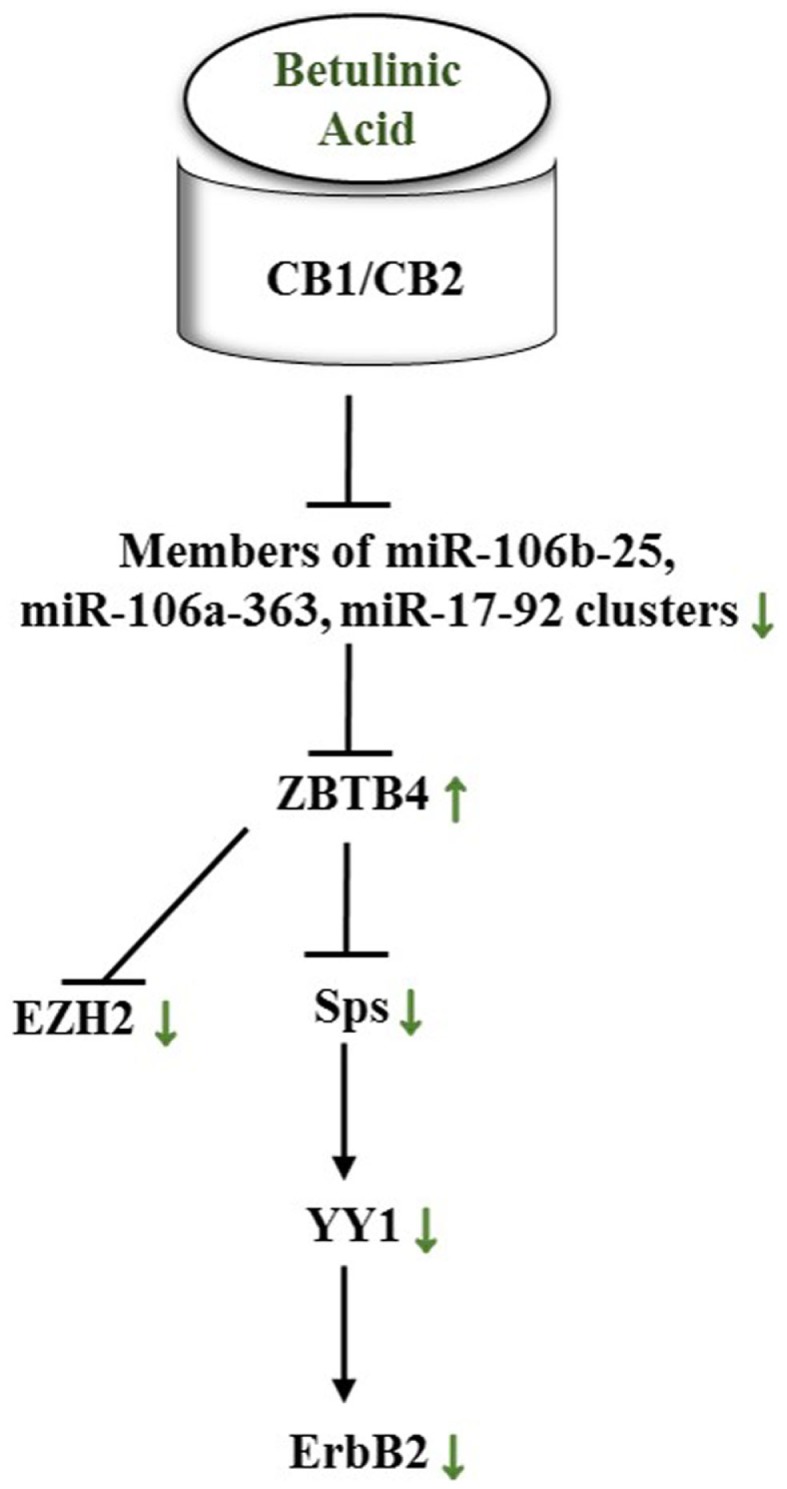
**Betulinic acid impacts a miR-ZBTB4-Sp-axis in female cancers through CB1/CB2 receptors**. Green arrows show actual impact of betulinic acid on targets in female cancer models. See text for indicated changes, as each presented axis represents findings illustrated in the text.

Alongside pomegranate polyphenols, betulinic acid, a pentacyclic triterpenoid rife in barks of trees such as *Betula pubescens* as well as rosemary, is able to decrease miR-27a levels in MDA-MB-231 BC cells. Thus, the same axis targeted by pomegranate polyphenols is affected by betulinic acid (Figure 7). In the according study, the group established the fact that betulinic acid nullified the angiogenic, proliferative phenotype caused by Sps overexpression not only through their repression but also by reducing vascular endothelial growth factor (VEGFR) mRNA, down-regulation of miR-27a, and consequently Myt-1 up-regulation. Myt-1 in turn catalyzes the repressive phosphorylation of cdc2 and blocks in this manner G_2_/M cell cycle progression (149). Moreover, Yang et al. reported a disruption of an oncogenic miR-ZBTB4-Sp axis. Similar to ZBTB10, ZBTB4 is repressed by miRs, namely miR-20a, miR-106a, and miR-106b from the miR-17-92, miR-106a-363, and miR-106b-25 clusters. Betulinic acid at 15 μM effectuated a higher abundance in ZBTB4 and a lower abundance in Sp1, Sp3, Sp4, EZH2, miR-106a, miR-106b, and miR-20a. In MDA-MB-231 xenografts in athymic nude mice, 15 mg/kg/day of betulinic acid showed the same results (150). ERBB2 overexpressing BT474 and MDA-MB-453 BC cells revealed in a further study that treatment with betulinic acid in the range of 1–10 μmol/L resulted in a decrease of Sp1, 3, and 4, and consequently in a decrease of YY1 promoter activity as well as a decrease in ERBB2. It was moreover discovered that the impact of betulinic acid on the aforementioned axis was cannabinoid 1 (CB1) and CB2 receptor-dependent (151) (Figure 8).

#### 1′S-1′-Acetoxychavicol Acetate

In addition to the DIM-Herceptin synergism, an interesting observation was made by Phuah et al. *Alpinia conchigera*, also known as wild ginger, contains the phytochemical 1′S-1′-acetoxychavicol acetate (ACA). ACA acts, when administered before or together with Cisplatin, synergistically in Ca Ski and HeLa cells and may provide a possible future therapeutic approach of cervical carcinoma treatment. To determine the induced changes in global miR levels, microarray studies were conducted. These revealed in total 25 miRs differentially expressed due to ACA and/or Cisplatin treatment. ACA alone induced up-regulation of miR-629, 487a, 483-3p, 376a, 342-3p, and 212 expression, while the expression of miR-1262, 875-3p, 517, and 411 was down-regulated due to ACA treatment. A combinatorial approach with Cisplatin resulted in increased levels of miR-922, 744, 523, 210, and 138; miR-1271, 224, and 21 levels in turn were decreased. *In silico* analyses furthermore suggested for miR-138, miR-210, and miR-774 an involvement in Wnt, ERK, NFκB, TGF-β, and Ca2+ signaling. ACA embodies a promising chemosensitization agent for abating cellular glutathione levels, which can prevent Cisplatin inactivation, DNA repair, and reduced oxidative stress in cancer cells. Importantly, it antagonizes Cisplatin when the cells were pre-treated with Cisplatin. This notion sheds valuable information on feasible future combinatorial therapy approaches (152).

### Garcinol

Garcinol, a polyisoprenylated benzophenone derivative found in *Garcinia indica* also known as kokum, is utilized broadly in Indian cuisine and Indian traditional medicine. It has pleiotropic effects on various diseases, such as cancer. Ahmad et al. were the first to establish that garcinol induced cancer cell-specific apoptosis and MET through down-regulation of NFκB p65, modulation of miRs, and inhibition of Wnt signaling. miR-200s were shown to be an opponent of the p65 subunit of NFκB, the intercept point of multiple signaling pathways. These results were based on the discovery that garcinol induced down-regulation of the mesenchymal markers vimentin, ZEB-1, and ZEB-2 and also up-regulation of the epithelial marker E-cadherin. Garcinol furthermore significantly increased the expression of miR-200b, miR-200c, and the let-7 family members let-7a, let-7e, let-7f. miR-200 and let-7 family have been propounded to maintenance and regulation of EMT/MET. A connection between the Wnt pathway and miR-200s is constituted through the fact that miR-200a may target β-catenin. Taken together, the *in vitro* and *in vivo* findings on garcinol’s impact ought to be enlarged. It is of particular interest whether synergism with other nutraceuticals or chemotherapeutics in female cancers exists. Curcumin and gemcitabine for instance were already tested and verified in studies in pancreatic cancer, but synergistic pairs involving garcinol are not identified in breast and gynecological cancers (153, 199–201).

### Glyceollins

Soy plants grown in stress conditions produce a large quantum of the phytoalexins glyceollin I–III. *In vivo* glyceollins withhold the tumor formation of ER+ and estrogen-dependent BC models by exhibiting an anti-estrogenic effect. Following the notion that glyceollins may affect ER− cancer, the impact of glyceollins on the TNBC cell lines MDA-MB-231 and -468 was studied. This assumption was confirmed when treatment of mice with xenografts resulted in partial tumor growth suppression. In MDA-MB-231 cells treated with 10 μM of glyceollins for 18 h, significant changes in the miRnome and the proteome were observed. miR-181c/d involved in cell cycle arrest and inhibition of proliferation; miR-22, 29b/c, 30d, 34a, and 195 associated with suppression of EMT and metastasis in breast/other cancers as well as miR-26b targeting oncogenes directly were highly expressed upon treatment. In reference to their functions and effects, miR-22 targets the pro-metastatic EZR in OC and oncogenes EVI-1, ERBB2, CDC25 in metastatic BCs. Glyceollin-induced miR-26b elevation suppresses SLC7A11, thus leading to apoptosis in BC samples, while miR-29b and c seem to hold an ambivalent character. In HeLa and HFF cells, miR-29b/c induces tumor cell senescence, while they are members of an up-regulated miR group that has been associated with a poor prognosis in BC samples. Similar to resveratrol, miR-663 was up-regulated by glyceollins. Concerning the putative oncomiRs involved in cancer progression, miR-193a-5p, 197, 224, 486-5p, and 542-5p were down-regulated by glyceollins. The latter were suggested to target non-metastatic cells 1 (NME1), a metastasis suppressing gene. Thus, glyceollins-dependent decrease of 486-5p and 542-5p led to a significant increase of NME1. In contrast to miR-193a-5p, 197, and 486-5p, which have not been ascribed a role in female malignancies yet^1^, miR-224 was differentially expressed in OC and miR-542-5p up-regulated in the mesenchymal phenotype compared to the epithelial phenotype of endometrial carcinosarcoma (42, 154, 202–204).

### Matrine

Present studies on the alkaloid matrine from *Sophora flavescens*, an evergreen shrub of the Fabaceae family, commonly employed in Chinese medicine, reveal inhibitory effects on BC cell division, migration, and metastasis *in vitro* and *in vivo*. As a consequence, matrine was utilized as adjuvant therapy to enhance 5-year survival rate of mastocarcinoma patients in China. Although little is known about matrine’s molecular effects, Li et al. investigated this in MCF-7 BC cells. MTT assays indicated an IC_50_ of ~0.8 mM for 48 h. Akin to curcumin, genistein, and resveratrol, matrine targets the miR-21/PTEN/AKT axis in cellular signaling of BC cells. Matrine treatment resulted in down-regulation of miR-21, accordingly an augmentation of PTEN protein and dephosphorylated AKT; therefore, downstream targets of AKT such as BAD (dephosphorylated), p21, and p27 (up-regulated) were affected as well (155).

### Artemisinin and artesunate

The phytochemical artemisinin, a compound found in the sweet wormwood plant, and its derivative artesunate, have been studied as compounds against BC recently. Exhibiting potent anti-malarial activity, artemisinin and its derivative artesunate have a peroxide moiety that can react with iron resulting in the formation of free radicals. Due to the fact that cancer cells hold more intracellular free iron, these compounds act cancer-cell specific (205). In a study of Hargraves et al., it was shown that these compounds induce cell-cycle arrest in MCF7 and T47D BC cells. Furthermore, artemisinin and its derivative were able to suppress CDK4 through up-regulation of miR-34a in a p53 independent manner, as opposed to the aforementioned compound I3C (145). Owing to their short plasma-half life, more potent analogs are under investigation (205).

### Vitamins

Although vitamins are not primarily considered phytochemicals, a part of these are essential nutrients with a demonstrable impact on miR signatures in breast and gynecological cancers. One of them is ascorbic acid (vitamin C). Ascorbic acid up-regulates expression of NRF2 as well as NRF2- related genes, superoxide dismutase and NAD(P)H:quinone oxidoreductase, by decreasing miR-93 levels in MCF10A and T47D cells. Furthermore, reduced levels in NRF2 due to an increase of miR-93 were reversed upon vitamin C treatment in an E2-induced ACI rat model. In MCF10A cells, miR-93 suppression promoted a decrease in colony and mammosphere formation as well as apoptosis induction. These findings highlight not only a further mechanism through which diet can influence cancer but also a possible combinatorial approach of cancer treatment in the future (156).

Vitamin D_3_ occurs in several forms, such as calcitriol and calcifediol. Both are synthesized by the body itself and exert hormonal function; nevertheless, supplementing vitamin D_3_ in 30–80 ng/ml doses were determined as beneficial in terms of carcinogenesis. 1, 25-dihydroxyvitamin D_3_ (calcitriol) acts through the vitamin D receptor (VDR) as a modulator of the immune system, cancer cell proliferation, and apoptosis. Ligand binding to the VDR is followed by heterodimerization with the retinoid X receptor (RXR) and consequently interaction with vitamin D-responsive elements in the regulatory region of according genes. Kasiappan et al. substantiated that in response to calcitriol treatment, not only *hTERT* mRNA stability decreased through increment of miR-498 expression within 30 min of 10^−7^M calcitriol (157). These results may deem the miR-498 gene as an immediate response toward the treatment.

Because cholecalciferol may confer toxicity in terms of evoking hypercalcemia, the prohormone calcifediol has gained attention as it is able to protect against cellular stress, such as hypoxia or induction of ROS, a risk factor for developing cancer. In a study performed with non-malignant MCF12F breast epithelial cells, five miRs (miR-26b, miR-182, miR-200c, miR-200b, and let-7b) have been identified to be comprised in the cellular stress response (158). Consecutively, the highest increased miR, miR-182, having two binding sites for p53, was shown to be decreased by calcifediol and suppressed cell proliferation when overexpressed in MCF12F cells. These results may indicate that dietary supplementation with calcifediol can contribute to chemoprevention (157, 158, 206). Table 2 gives an overview of the miRs targeted and altered by phytochemicals in female cancers.

## Conclusion and Future Perspectives

ClinicalTrials.gov^2^ listed 407 studies to date and counting on the effect of diet on female cancers revealing the ubiquitous understanding that nutrition can have a beneficial influence on cancer prevention and treatment. This is supported by the plethora of findings which substantiate that nutraceuticals are important in primary, secondary, and tertiary chemoprevention as well as in combination with chemotherapeutic agents. Reactivation of ERα or enhancing the effect of Cisplatin, Herceptin, and various other chemotherapeutics implies the necessity of a combinatorial therapeutic approach for cancer therapy. Therefore, the all-embracing understanding of the molecular mechanisms induced by these compounds is not only salutary but inevitable: examining miR patterns and their associated phenotypes in response to phytochemical treatment is a central approach.

In fact, little is known on the structure-activity relationship of phytochemicals with miRs in female cancers. Major open questions, such as in which manner phytochemicals can bind to miRs or their genes, as well as mechanistic insights into feed-back loops or pathways leading to the phytochemical-induced miR level alteration, remain. First findings reveal that p53 status may be a key factor for some compounds. In this connection, patients with certain tumor characteristics, such as a p53 negative status, may not benefit from a compound that is only effective in patients with p53 wild-type positive cancers (145). Hence, establishing pathways leading to phytochemical-dependent miR level changes along with further research on comparatively novel substances such as brusatol or artemisinin is necessary. Knowledge on whether these substances can act synergistic or antagonistic could enhance treatment as well as cancer prevention tremendously.

As indicated before, a future perspective is the co-administration of chemotherapy with a single or a combination of phytochemicals. Chemotherapy is administered depending on the patients’ tumor characteristics; however, there is a lack of knowledge about which cancer genotype as well as phenotype will benefit from additional phytochemical treatment. Moreover, little is known about the interaction between phytochemical and chemotherapy agents. As emphasized before, DIM and Herceptin; ACA and Cisplatin may be efficient combinations for therapy of female cancers. Sulforaphane has been reported to enhance drug cytotoxicity of various chemotherapeutics in prostate and pancreatic CSC, if this may apply to female cancers and the according CSCs as well will need further investigations (207). On the other hand, curcumin as well as vitamins have been reported to interfere with chemotherapy, thus answering the question in which manner phytochemicals affect treatment is crucial (170, 207). In the future, elucidating clinical significances of the co-administration of phytochemicals is an important field of study and ought to focus on further *in vivo* studies as well as clinical trials with an according trial design. In this respect, not only the established biomarkers but the individual miR levels may play a significant role in the outcome of the patient and the trial.

Yet, when it comes to dealing with miR profiling studies, there are currently limitations in terms of the outcome. Nair et al. showed in their analysis of 43 miR profiling studies that significant interstudy irregularities concerning the amount of cohorts/samples and external validations existed among the studies. This suggests to remit guidelines in order to bring different studies down to a common denominator and to facilitate assessment of varying inter-study results achieved in the future (8). A further important aspect is the extension of studies dedicated toward the impact of phytochemicals on miR levels in female cancer, as miR-directed therapies may be an important approach. First promising results of miR-directed therapies are evident in the field of hepatitis c virus research. In this connection, finding antimiRs with the ability to scale down tumor aggressiveness, for instance, is of great interest (8). Because the delivery of non-coding RNA targeting therapeutics is not yet at an appropriate standard, supplementary studies addressing possible strategies such as nanoparticles, liposomes, or viral carriers ought to be conducted.

Excitingly, there has been a mechanism leading to transcriptional activation known as RNA activation (RNAa) described. Through targeting promoter sequences by double-stranded saRNA (small activating RNA), gene expression was activated. Further investigations on how saRNA or rather miRs can manipulate cell fate as well as screening for endogenous activating RNAs in humans is necessary. Huang et al. demonstrated that in mouse cells an endogenous system exists, activating gene expression through miRs (208, 209). Hence, finding putative RNAas induced by natural agents may open splendid new avenues for cancer treatment. These may comprise the activation of silenced or down-regulated tumorsuppressor genes coding for miRs or other anti-tumor entities.

In conclusion, phytochemicals exert their anti-inflammatory, anti-oxidant, and anti-cancer effects along with a variety of other functions not only through targeting epigenetic modulators such as HATs, HDACs, and DNMTs but also through targeting miRs, which are feasible for influencing these aforementioned modulators as well as further cellular signaling cascades. Not only phytochemicals such as curcumin, genistein, or resveratrol but vitamin C, D3, and polyunsaturated fatty acid from fish oil have been shown to impact miR patterns in female cancers. Hence, including phytochemicals in treatment is likely to enhance the outcome of diseases such as female cancers at minimum risk. As more data become available, knowledge about miR profiles and their implication converges with medicine and personalized treatment (156–158, 210). The fact that Western countries tend to a higher incidence in cancers, such as female cancer, highlights the importance of molecular research on dietary components and health-conscious nutrition. Understanding the comprehensive picture of cellular pathways, including miRs and how these pathways can be utilized to prevent and treat female cancer, is an important goal. Because phytochemicals have a low risk of severe side effects, employing these in addition to chemotherapeutic agents high in side effects can be extremely valuable once administered correctly. Using the nutraceuticals tool in the correct way may significantly prevent cancer or enhance therapy outcomes for patients tremendously.

Considering the presented aspects and approaches, the knowledge on how nutrition significantly changes or interferes in cross-talk among pathways of miRs is a matter of great prominence, especially for developing new treatments targeted at low treatment-responsive female cancers.

## Conflict of Interest Statement

The authors declare that the research was conducted in the absence of any commercial or financial relationships that could be construed as a potential conflict of interest.

## Footnotes

^1^http://mir2disease.org/

^2^https://clinicaltrials.gov/
